# Plant-derived bioactives, the gut–brain axis, and neurodegenerative diseases: mechanistic roles of diet–microbiota interactions

**DOI:** 10.3389/fnins.2026.1815972

**Published:** 2026-05-28

**Authors:** Ashley Reynolds, Ethan Glenn, Brigitte Lavoie, Suzanne L. Ishaq, Yanyan Li

**Affiliations:** 1 School of Food and Agriculture, University of Maine, Orono, ME, United States; 2 School of Pharmacy and Pharmaceutical Sciences, SUNY Binghamton University, Binghamton, NY, United States; 3 Department of Neurological Sciences, University of Vermont College of Medicine, Burlington, VT, United States

**Keywords:** cruciferous vegetables, glucoraphanin, gut-brain axis, microbial metabolites, neurodegenerative diseases, neuroinflammation, plant-based nutrition, sulforaphane

## Abstract

Diet is increasingly recognized as a potential upstream modulator of the gut-brain axis (GBA) through its effects on the microbiome, microbial metabolites, and host immune and endocrine responses. The GBA is a complex, bidirectional network connecting the gastrointestinal tract and central nervous system, with diet influencing microbial community structure and metabolic output. Plant-based diets, such as Mediterranean and MIND, have been associated with increased production of anti-inflammatory microbial metabolites and improved barrier function, while high calorie/low nutrient diets are often linked to increased immune activation and barrier dysfunction. However, while microbial metabolites, especially short-chain fatty acids, indoles, bile acids, and isothiocyanates, have been proposed as mediators of neuroprotective effects, their role in neurodegenerative diseases remains an area of active investigation, with evidence largely derived from preclinical and associative human studies. Cruciferous vegetables, especially broccoli sprouts, are an emerging focus of research for their bioactive compound sulforaphane, which activates Nrf2-centered cytoprotective pathways. Animal and early human studies suggest sulforaphane can improve cognitive and behavioral outcomes, though larger clinical trials are needed. Personalized, microbiota-targeted dietary interventions may offer scalable strategies for managing neuroinflammatory and neurodegenerative conditions, and we emphasize the need for integrated research across diet, microbiome, and brain health.

## 1 Introduction

Health, diet, and the gut microbiome are intricately connected, and growing evidence suggests that dietary components can shape neurological outcomes through their effects on microbial metabolism and immune signaling (Loh et al., 2024). The gut microbiota is a complex microbial ecosystem comprised of bacteria, fungi, viruses, and occasionally protozoan parasites, and is well known to influence host nutrition as well as numerous physiological processes of the host, including anatomical and physiological development, immune regulation, and responses to stress or aging (Taraskina et al., 2022; Warren et al., 2024). While prior reviews have examined the gut microbiota’s role in mediating gut-brain communication, this review synthesizes current mechanistic and translational evidence linking dietary patterns and bioactive phytochemicals to microbiota-derived metabolites, immune activity, and neurochemical signaling within the gut-brain axis (GBA). Importantly, although these pathways provide biologically plausible links to neuroinflammation and neurodegenerative disease, much of the evidence remains associative or derived from preclinical models, and causal relationships in humans are still being established (Box 1).

BOX 1 **Key open questions and limitations**

• Much of the field remains based on preclinical and associative human evidence rather than established causality

• Microbial taxa and metabolites show strong context dependence, making simple “beneficial” versus “harmful” classifications inadequate

• Barrier dysfunction, immune activation, and altered metabolite signaling are strongly implicated, but their temporal and causal roles in human disease remain uncertain.

• Dietary intervention studies are promising but are often limited by small sample sizes, short duration, and heterogeneous outcome measures.

• Sulforaphane represents a distinctive and mechanistically tractable example, but human evidence remains early and questions of bioavailability, dosing, and microbiota-dependent activation remain unresolved.

Future progress will require longitudinal, well-controlled human studies integrating diet, microbiome function, metabolomics, immune profiling, and neurological outcomes.

### 1.1 Gut-brain axis pathways

The GBA is a bidirectional communication network connecting the gastrointestinal (GI) tract and the central nervous system (CNS), integrating neurological, immune, and metabolic processes implicated in a wide range of neurodevelopmental and neurodegenerative conditions, including Parkinson’s disease (PD), Alzheimer’s disease (AD), multiple sclerosis (MS), depression, and autism spectrum disorder (ASD) (Bonaz et al., 2018; Taraskina et al., 2022; Yuan et al., 2023; Loh et al., 2024). GBA communication is mediated through multiple, interconnected pathways, including the vagus nerve (Cranial nerve X), immune signaling, barrier modulation, and the enteric nervous system (ENS), with diet and microbial metabolites (Table 1) serving as shared molecular mediators across these routes (Warren et al., 2024). Diet interfaces with each of these pathways by shaping microbial community structure, metabolic output, and host nutrient and hormone signaling (Tables 2, 3).

**Table 1:** Key microbial metabolites linking diet, microbiota, and CNS outcomes.

Metabolite class	Source (diet/microbes)	Primary mechanisms	Effects on the CNS/GBA	Study type	Disease relevance	Reviewed in
Short-chain fatty acids	Dietary fiber fermented by gut microbes	FFAR2/FFAR3 signaling; HDAC inhibition; energy substrate for colonocytes; reinforcement of intestinal and BBB tight junctions	Reduced neuroinflammation; support of microglial maturation and immune homeostasis; improved BBB integrity	Preclinical; indirect human evidence	AD, PD, MS; cognitive aging	Abdel-Haq et al. (2019), Bostick et al. (2022), Braniste et al. (2014), Erny et al. (2015), Kelly et al. (2015), Parada Venegas et al. (2019), Parker et al. (2019), Yadav et al. (2022), Yan and Ajuwon (2017), and Yang et al. (2024)
Tryptophan-derived indoles	Dietary tryptophan metabolized by gut microbes	AhR and PXR signaling; modulation of immune and epithelial responses; indirect vagal activation via enteroendocrine cells	Context-dependent suppression or exacerbation of neuroinflammation; modulation of microglial phenotypes; altered autonomic gut–brain signaling	Preclinical; limited human associative evidence	MS, AD; CNS autoimmunity	Ashique et al. (2024), Coretti et al. (2024), Jameson et al. (2025), Montgomery et al. (2024a,b), and Sittipo et al. (2022)
Bile acids	Host-derived bile acids (primary) modified by gut microbes (secondary)	TGR5 and FXR signaling; enteroendocrine hormone release (GLP-1, FGF19); direct CNS receptor engagement	Modulation of neuroinflammation; effects on BBB permeability; endocrine and vagal gut–brain signaling	Preclinical and human associative evidence	AD, PD	Ferrell and Chiang (2021), Mertens et al. (2017), Montgomery et al. (2024a,b), and Sittipo et al. (2022)
Polyphenol-derived microbial metabolites	Dietary polyphenols biotransformed by gut microbes	Antioxidant and anti-inflammatory signaling; mitophagy induction; modulation of microglial activity	Reduced oxidative stress; attenuation of neuroinflammation; neuroprotection in disease models	Preclinical; emerging human evidence	AD, PD, MS	Fujisaka et al. (2023) and Rowland et al. (2018)
Isothiocyanates (e.g., sulforaphane)	Cruciferous vegetables; microbial and plant myrosinase conversion	Nrf2/ARE activation; suppression of NF-κB, MAPK signaling; epigenetic regulation (HDAC/DNMT inhibition)	Reduced microglial inflammation; improved barrier integrity; antioxidant and cytoprotective effects; improved cognitive and behavioral outcomes	Preclinical; early clinical and pilot trials	AD, PD, MS, stroke, depression	Alaba et al. (2024), Bessetti and Litwa (2025), Eren et al. (2018), Han et al. (2017), He et al. (2022), Holcomb et al. (2023), Holman et al. (2023), Houghton (2019), Kamal et al. (2022) Kapoor et al. (2022), Santín-Márquez et al. (2019), Schepici et al. (2020), Subedi et al. (2019), Tang et al. (2022), Zhao et al. (2006), and Zhao et al. (2018)
Serotonin-related compounds	Host synthesis regulated by microbial metabolites; limited microbial production	Microbial induction of TPH1 in enterochromaffin cells; ENS and vagal signaling	Indirect modulation of mood, stress responses, gut motility, and gut–brain communication	Preclinical; limited human evidence	AD, PD; mood disorders	Gershon and Margolis (2021), Moretti et al. (2025), Roth et al. (2021), and Yano et al. (2015)
Dopamine-related metabolites / precursors	Microbial regulation of tyrosine and L-DOPA metabolism	Regulation of precursor availability; modulation of peripheral dopamine metabolism	Influence on dopaminergic signaling and L-DOPA responsiveness	Mostly preclinical	PD	Daubner et al. (2011) and Hamamah et al. (2022)
GABA (*γ*-aminobutyric acid)	Produced by select gut microbes from glutamate	Microbial GABA synthesis; vagal and immune modulation	Reduced CNS excitability; modulation of mood and stress-related behaviors	Preclinical; small human trials	Anxiety, depression; neurodegeneration	Braga et al. (2024), Casertano et al. (2024), Hills et al. (2019), Miri et al. (2023), Patterson et al. (2019), and Yan and Ajuwon (2017)
Glutamate-related metabolites	Diet-dependent amino acid metabolism shaped by microbiota	Regulation of glutamine–glutamate cycling; excitatory or inhibitory balance	Modulation of excitotoxicity risk and neuronal vulnerability	Preclinical	AD, PD	Gruenbaum et al. (2024), Ma et al. (2018), Warren et al., (2024), and Wu et al. (2022)

**Table 2:** Dietary patterns and GBA outcomes in clinical studies.

Dietary pattern	Key features (human studies)	Reported microbiota/metabolite effects	Neurological or GBA outcomes observed in humans	Reviewed in
Mediterranean diet	High fruits, vegetables, whole grains, legumes, nuts, olive oil	Increased microbial diversity; increased SCFAs and anti-inflammatory metabolites	Associated with reduced neuroinflammation and improved cognitive function; protective associations in AD and PD	Ayten and Bilici (2024)
MIND diet	Mediterranean + DASH elements; emphasis on leafy greens, berries	Promotes saccharolytic fermentation and SCFA production	Linked to reduced cognitive decline and neuroinflammation via microbiota-dependent mechanisms	Ayten and Bilici (2024)
Vegetarian/low-protein, high-carbohydrate diets	Reduced animal protein; plant-forward	Shifts microbiota toward saccharolytic metabolism; reduced proteolytic metabolites	Modest improvements in motor symptoms, GI function, and inflammatory markers in some PD cohorts	Ayten and Bilici (2024)
Ketogenic diet (KD)	High fat, very low carbohydrate	Alters microbiota toward ketone-utilizing taxa	May support cognitive function in individuals with mild cognitive impairment	Ayten and Bilici (2024)
Mediterranean ketogenic diet (MMKD)	Ketogenic macronutrients with Mediterranean food sources	Combines ketone metabolism with microbiota-supportive foods	Cognitive benefits reported in mild cognitive impairment	Ayten and Bilici (2024)
Cruciferous vegetable–rich diets (e.g., broccoli sprouts)	Glucosinolate-rich foods producing sulforaphane	Microbiota-dependent conversion to isothiocyanates	Early human studies suggest improvements in cognitive or behavioral outcomes and reduced oxidative/inflammatory markers	Houghton (2019) and Schepici et al. (2020)

**Table 3:** Dietary patterns and mechanistic GBA effects in animal and preclinical models.

Dietary pattern/exposure	Model systems	Microbiota or metabolite effects	Mechanistic or neurological outcomes	Reviewed in
Fiber-rich, plant-based diets	Rodent	Increased SCFAs; improved microbial stability	Enhanced barrier integrity, reduced immune activation, improved vagal signaling	Bonaz et al. (2018), Coretti et al. (2024), and Janakiraman and Krishnamoorthy (2018)
Western-style diet	Rodent and in vitro	Reduced SCFAs; increased LPS and secondary bile acids	Increased gut permeability, immune activation, and neuroinflammation	Coretti et al. (2024) and Janakiraman and Krishnamoorthy (2018)
High-salt diet	Rodent	Promotes pro-inflammatory immune signaling	Worsened disease severity in MS-like models	Coretti et al. (2024)
Ketogenic diet (KD)	Rodent	Alters microbial composition and metabolite profiles	Modulation of neuroinflammation and brain energy metabolism	Ayten and Bilici (2024)
Cruciferous vegetables/sulforaphane	In vitro and multiple rodent models (EAE, PD, AD, stroke, depression)	Microbial and enzymatic conversion to sulforaphane; Nrf2 activation	Reduced neuroinflammation, improved barrier integrity, antioxidant and cytoprotective effects, improved behavioral outcomes	Alaba et al. (2024), Bessetti and Litwa (2025), Eren et al. (2018), Han et al. (2017), Holcomb et al. (2023), and Holman et al. (2023)

The vagus nerve directly relays afferent sensory signals from the gut to the brain, and efferent motor commands from the brain to the GI system, thereby regulating autonomic, inflammatory, and neuroendocrine processes that influence mood, immune function, and stress response (Warren et al., 2024). Although vagal afferents do not directly contact the gut microbiota, they detect diet-shaped microbial metabolites, such as short-chain fatty acids (SCFAs) via Free Fatty Acid Receptor 2 (FFAR2) and Free Fatty Acid Receptor 3 (FFAR3), and secondary bile acids (BAs) via Takeda G protein-coupled bile acid receptor 1 (TGR5), through epithelial, enteroendocrine, and immune intermediaries (Warren et al., 2024). In contrast, tryptophan-derived indoles, including indole-3-propionic acid and 3-indoxyl sulfate, primarily signal indirectly through epithelial and immune receptors such as Aryl hydrocarbon receptor (AhR) and Pregnane X receptor (PXR), rather than through direct vagal binding (Warren et al., 2024). Antigens can also be sensed indirectly through interactions with enteroendocrine cells and immune cells in the intestinal epithelium, which can sense bacterial products such as lipopolysaccharide (LPS) through toll-like receptors (TLRs) and communicate those signals to the vagus nerve (Janakiraman and Krishnamoorthy, 2018). In response to microbial signals or disruption of a stable and functional microbial community, immune cells release pro-inflammatory cytokines and chemokines that influence blood–brain barrier (BBB) function, either directly or via activation of vagal afferent fibers that relay immune signals to the brain (Janakiraman and Krishnamoorthy, 2018). Chronic activation of immune pathways involving the vagus nerve has been associated with increased neuroinflammatory signaling and elevated risk for neurodegenerative and psychiatric disorders, although much of this evidence is derived from preclinical and observational studies (Janakiraman and Krishnamoorthy, 2018). Reduced vagal tone, which is often observed in patients with irritable bowel syndrome (IBS) and inflammatory bowel disease (IBD) is associated with increased intestinal permeability and systemic inflammation (Janakiraman and Krishnamoorthy, 2018). Dietary patterns that support microbial stability and the production of beneficial metabolites, such as fiber-rich and plant-based diets, may also support vagal signaling (Bonaz et al., 2018).

Immune signaling represents a major pathway through which gut-derived cues influence CNS function within the GBA (Yuan et al., 2023). In parallel with vagal-mediated immune sensing, the gut-associated lymphoid tissue (GALT) continuously samples microbial and dietary antigens and coordinates local and systemic immune responses (Yuan et al., 2023). Alterations in gut microbial composition can increase exposure to microbial products such as LPS, activating peripheral immune cells and promoting cytokine and chemokine release that may influence neural and glial signaling within the CNS (Yuan et al., 2023). These immune mediators may affect brain function either by crossing a compromised BBB or by indirectly modulating neural and glial signaling pathways (Yuan et al., 2023). Peripheral immune activation has been shown, particularly in preclinical models, to contribute to neuroinflammatory processes by promoting infiltration of immune cells, including macrophages and T cells, into the CNS, where they interact with resident glial populations and amplify inflammatory signaling (Ma et al., 2019). Gut dysbiosis may further contribute to this process by increasing pro-inflammatory mediators (LPS and secondary BAs) while reducing the availability of anti-inflammatory SCFAs that normally support epithelial barrier function and immune homeostasis (Ojeda et al., 2021; Bostick et al., 2022). While alterations in barrier integrity and immune activation are frequently associated with neuroinflammatory states, direct causal links between gut or BBB dysfunction and disease progression in humans remain incompletely established. These changes bias immune signaling toward sustained activation through pattern-recognition receptors such as TLRs (Bostick et al., 2022). Within the CNS, microglia serve as primary immune responders and integrate peripheral inflammatory signals (Ojeda et al., 2021). Chronic immune activation promotes microglial phenotypes characterized by elevated cytokine production, oxidative stress, and impaired resolution of inflammation, contributing to synaptic dysfunction and neurodegeneration in conditions such as AD and PD (Janakiraman and Krishnamoorthy, 2018; Gasmi et al., 2022; Gao et al., 2023). Emerging evidence indicates that microglial responses exist along a spectrum of activation states, with context-dependent roles in aging and disease progression (Ayten and Bilici, 2024).

Barrier integrity is a critical structural regulator of the GBA communication, governing the extent to which microbial products and immune mediators access systemic circulation and the CNS (Yang et al., 2024). The intestinal epithelial barrier, composed of tightly regulated junctional proteins (including occludin, claudins, and zonula occludens proteins) and a protective mucus layer, limits translocation of luminal microbes and endotoxins under homeostatic conditions (Bostick et al., 2022; Yang et al., 2024). Disruption of this barrier, driven by gut dysbiosis, inflammatory dietary patterns, or microbial products such as LPS and secondary BAs, increases intestinal permeability and promotes systemic immune activation (Ojeda et al., 2021; Bostick et al., 2022). Gut and brain barrier systems are functionally interconnected within the GBA. Loss of intestinal barrier integrity enhances peripheral inflammation that destabilizes BBB tight junctions through cytokine signaling, oxidative stress, and matrix metalloproteinase activation, thereby facilitating immune cell and cytokine entry into the CNS (Yang et al., 2024). Conversely, microbial metabolites (Table 1) such as SCFAs, particularly butyrate, support both intestinal and BBB integrity by reinforcing tight junction expression, enhancing mucus production, and promoting regulatory immune responses (Bostick et al., 2022; Yang et al., 2024). Coordinated disruption of gut and brain barriers therefore creates a permissive environment for sustained immune-neural crosstalk, linking peripheral microbial perturbations to chronic neuroinflammation and neurodegenerative disease progression (Ojeda et al., 2021; Yang et al., 2024). Notably, alterations in gut microbial composition, barrier function, and microbial metabolic output have been detected prior to the onset of overt neurological symptoms in several neurodegenerative diseases (Ojeda et al., 2021). In PD, GI dysfunction frequently precedes motor and cognitive impairments, supporting the concept that early gut-derived disturbances may contribute to disease initiation and progression (Loh et al., 2024).

The connection between the CNS and ENS is another important pathway for GBA. Sometimes referred to as the “second brain,” the ENS is an extensive network of neurons embedded in the gut wall that independently regulates many aspects of GI motility, secretion, and local blood flow (Carabotti et al., 2015; Jaberi et al., 2024). Through neurotransmitters and neuropeptides, the ENS can influence mood, behavior, and stress responses. The ENS also interacts bidirectionally with the CNS through sympathetic and parasympathetic pathways and integrates neural, immune, and metabolic signals within the GBA (Jaberi et al., 2024). Disruptions in the communication between the ENS and the CNS have been implicated in disorders like IBS, PD, anxiety, and related conditions, underscoring the importance of gut-derived signals in shaping central neural function (Carabotti et al., 2015; Jaberi et al., 2024).

### 1.2 Microbiota-driven neurodegenerative disorders and cognitive aging

Building on the GBA pathways described above, the gut microbiota plays an important role in modulating neuroimmune interactions, particularly through its influence on neuroinflammation (Barberio et al., 2021). Neuroinflammation is a defining feature of many neurological disorders, including MS, PD, AD, stroke, and ASD (Barberio et al., 2021; Bessetti and Litwa, 2025; Carabotti et al., 2015). Microbial communities influence CNS immune responses via multiple mechanisms, including microbial metabolites, immune signaling pathways, and vagus nerve activity (Barberio et al., 2021). Evidence from germ-free and antibiotic-treated animal models has shown that disruption of the gut microbiota alters the severity of neuroinflammatory conditions (Barberio et al., 2021). Here, we synthesize evidence from human cohorts (Table 2) and animal models (Table 3) to illustrate how microbiota-driven immune dysregulation contributes to neuroinflammation, disease progression, and cognitive aging across neurological conditions.

Microbial community instability and loss of beneficial by-products are commonly observed in neurodegenerative and neurodevelopmental disorders (Bonaz et al., 2018; Bonfili et al., 2017). Human studies have found that in patients with MS, gut communities exhibit increased Akkermansia bacteria and Methanobrevibacter archaea and decreased Butyricimonas bacteria (Barberio et al., 2021). These changes are associated with altered immune responses. In PD, loss of bacterial commensals is associated with systemic inflammation, increased intestinal permeability, and early *α*-synuclein aggregation in the ENS which supports the hypothesis that PD may initiate in the gut (Eren et al., 2018; Erny et al., 2015; Feng et al., 2023). Similarly, post-stroke shifts in microbiota composition are accompanied by increased pro-inflammatory cytokines and impaired gut barrier integrity, suggesting that microbiota alterations contribute to immune dysregulation following ischemic injury (Bonaz et al., 2018; Bonfili et al., 2017).

New evidence indicates that gut microbiota contributions to MS arise from a three-way interaction among host genetics, microbial metabolism, and diet, rather than from discrete microbes acting independently (Ferrell and Chiang, 2021). Montgomery and colleagues demonstrated that genetic susceptibility loci shaping T cell signaling interacts with microbiota-derived immune-modulating signals, resulting in immune environments that either promote or suppress CNS autoimmunity (Ferrell and Chiang, 2021). Dietary patterns further shape this interaction, as high salt versus fiber-rich diets reshape the microbiota in ways that potentiate or dampen pro-inflammatory T helper 17 cell responses, which suggest that risk emerges through gene-diet-microbiome alignment rather than any single factor (Ferrell and Chiang, 2021).

Beyond overt neurological disease, gut microbiota composition has also been associated with cognitive aging and performance in older adults (Fujisaka et al., 2023). A large cross-sectional study found that individuals with decreased Firmicutes and Actinobacteriota and increased Bacteroidota, Proteobacteria, and Verrucomicrobiota, showed accelerated brain aging and reduced performance on cognitive tests like the MMSE and CDR-SB (Fujisaka et al., 2023). Mediation analysis showed that brain age significantly mediated the relationship between microbiota composition and cognitive function (Fujisaka et al., 2023). This suggests that gut microbiome composition may impact cognition by influencing neurodegenerative brain changes (Fujisaka et al., 2023). Importantly, these associations extend beyond overt neurological disease, implicating gut microbial function in normative cognitive aging trajectories.

A longitudinal MS cohort study showed that baseline microbiota profiles can predict future disability progression even in the absence of large diversity shifts (Gao et al., 2023). Patients who later worsened demonstrated lower abundances of *Akkermansia*, Lachnospiraceae, and Oscillospiraceae, and increased *Alloprevotella, Prevotella-9, Sutterella, Bilophila*, and Rhodospirillales (Gao et al., 2023). This suggests that microbiota-associated functional states, rather than taxonomy alone, act as early indicators of accelerated neurodegeneration and immune dysregulation (Gao et al., 2023).

In AD, microbial community shifts are linked to enhanced neuroinflammation, amyloid pathology, and gut barrier dysfunction (Gasmi et al., 2022). AD patients exhibit distinct microbial signatures which include a higher abundance of taxa like *Escherichia* and lower levels of beneficial taxa like *Roseburia* (Gasmi et al., 2022). Roseburia is associated with neuroprotective effects through the production of metabolites that support anti-inflammatory immune states (Gasmi et al., 2022). In contrast, *Escherichia* contributes to neuroinflammation through pathways that involve NLRP3 inflammasome activation and reactive oxygen species (Gasmi et al., 2022). These microbial shifts may contribute to M1 microglial activation, T-helper cell infiltration, and increased circulating endotoxins like LPS that can cross a leaky BBB and worsen inflammation (Gasmi et al., 2022).

In experimental autoimmune encephalomyelitis (EAE), a mouse model of MS, germ-free mice exhibit reduced disease severity, highlighting a central role for the gut microbiota in shaping neuroinflammatory disease progression (Barberio et al., 2021). Importantly, EAE studies further show that specific taxa, such as *Akkermansia muciniphila*, can exert either protective or pathogenic effects depending on microbial and dietary context (Gershon and Margolis, 2021). In some microbial community structures, *A. muciniphila* enhances pro-inflammatory Th17 cell responses and worsens CNS autoimmunity, while in others it promotes regulatory T cell-supportive environments that reduce disease severity (Gershon and Margolis, 2021). These findings help explain why some human MS studies report increased *Akkermansia* abundance and others find reduced levels in individuals with faster disease progression. Its functional impact appears to depend on the broader microbial environment in which it resides and dietary context rather than taxonomic presence alone (Gershon and Margolis, 2021). Continued research is needed to clarify context-dependent effects of microbial metabolites and define their roles as biomarkers or modulators of cognitive aging and neurodegenerative disease. These findings also highlight the importance of dietary factors, discussed in the following section, as potentially key modulators of microbiota composition and function within the GBA.

### 1.3 Effects of diet on the GBA

Dietary patterns are a primary upstream determinant of neuroinflammation, oxidative stress, and neuronal resilience, in part through their effects on the gut microbiota and its metabolic output (Janakiraman and Krishnamoorthy, 2018; Gasmi et al., 2022; Merlo et al., 2024). Diets rich in fiber and plant-based foods are associated with reduced neuroinflammatory responses, whereas Western-style dietary patterns high in salt and saturated fat exacerbate inflammatory pathways (Janakiraman and Krishnamoorthy, 2018). Specifically, high-salt diets have been shown to promote pro-inflammatory immune responses and worsen conditions like MS, whereas dietary interventions like increasing intake of non-fermentable fibers, reducing saturated fat, and adopting plant-based dietary patterns have demonstrated protective effects in experimental models of neuroinflammation (Coretti et al., 2024). Non-fermentable fiber supplementation has shown the ability to decrease intestinal BAs absorption and shift immune responses toward anti-inflammatory phenotypes (Janakiraman and Krishnamoorthy, 2018).

Diet represents the most direct and modifiable regulator of gut microbial composition and metabolic activity, with downstream consequences for neuroimmune signaling, barrier integrity, and gut-brain communication (Taraskina et al., 2022). Preventative and therapeutic dietary interventions that target the microbiota-gut-brain-immune interface show some promise for managing neuroinflammatory and neurodegenerative diseases (Warren et al., 2024). Dietary patterns like the Mediterranean and Mediterranean-DASH Intervention for Neurodegenerative Delay (MIND) diets (Table 2), which emphasize a high intake of fruits, vegetables, whole grains, legumes, nuts, and healthy fats, promote microbial diversity and increase production of anti-inflammatory microbial metabolites such as SCFAs, have been associated with reduced neuroinflammation and improved cognitive function (Ayten and Bilici, 2024). These diets offer anti-inflammatory and antioxidant benefits partly through microbiota-dependent mechanisms, and are considered protective against cognitive decline in both AD and PD (Ayten and Bilici, 2024). Vegetarian and low-protein with high-carbohydrate diets have both been explored in both observational and interventional studies, with findings suggesting modest and variable improvements in motor symptoms, GI function, and inflammatory markers in some, PD cohorts, in part by shifting gut microbial composition toward increased saccharolytic fermentation and reduced proteolytic metabolite production (Ayten and Bilici, 2024). Additionally, the ketogenic diet (KD), especially modified versions like the Mediterranean ketogenic diet (MMKD), has been associated with potential improvements in cognitive function in individuals with mild cognitive impairment (Table 2), possibly by targeting altered brain energy metabolism and modulating gut microbiome composition toward taxa associated with ketone utilization and reduced neuroinflammatory signaling (Ayten and Bilici, 2024). However, these findings are heterogenous and largely derived from small or short-term studies, and the sustainability and long-term effects of strict ketogenic diets remain uncertain (Ayten and Bilici, 2024).

Among dietary factors, phytochemicals from plant-based foods are increasingly recognized for their potential to modulate the GBA through anti-inflammatory and antioxidant properties (Jaberi et al., 2024). Plant-based dietary patterns provide both fermentable substrates that support microbial metabolism and a diverse array of phytochemicals that act in parallel with, and sometimes independently of, fiber-driven microbiota effects (Jaberi et al., 2024). Among these, polyphenols, such as flavonoids (e.g., quercetin) and stilbenes (e.g., resveratrol), represent some of the most extensively studied compounds and have been shown in preclinical and limited human studies to influence gut microbiota composition, immune signaling, and neuroinflammatory pathways (Jaberi et al., 2024). Curcumin and epigallocatechin-3-gallate (EGCG) have also demonstrated similar effects, particularly in *in vitro* and animal models, including modulation of inflammatory signaling and microbial community structure (Jaberi et al., 2024). While these findings support biologically plausible mechanisms linking phytochemicals to GBA function, the strength of evidence varies across compounds, and clinical data in humans remain limited and heterogeneous. The gut microbiota plays an important role in the metabolism and bioavailability of these phytochemicals and converts them into smaller and more bioactive metabolites (Table 1) that may cross the BBB and exert therapeutic effects (Fujisaka et al., 2023). The following sections synthesize evidence linking diet-microbiota interactions to neuroinflammation, cognitive aging, and neurodegenerative disease risk, emphasizing microbial metabolites as key mechanistic intermediaries connecting diet-driven changes in the microbiota to CNS outcomes (Kliewer and Mangelsdorf, 2015; Coretti et al., 2024).

## 2 Dietary factors and microbiota interactions in the GBA

**Figure 1:**
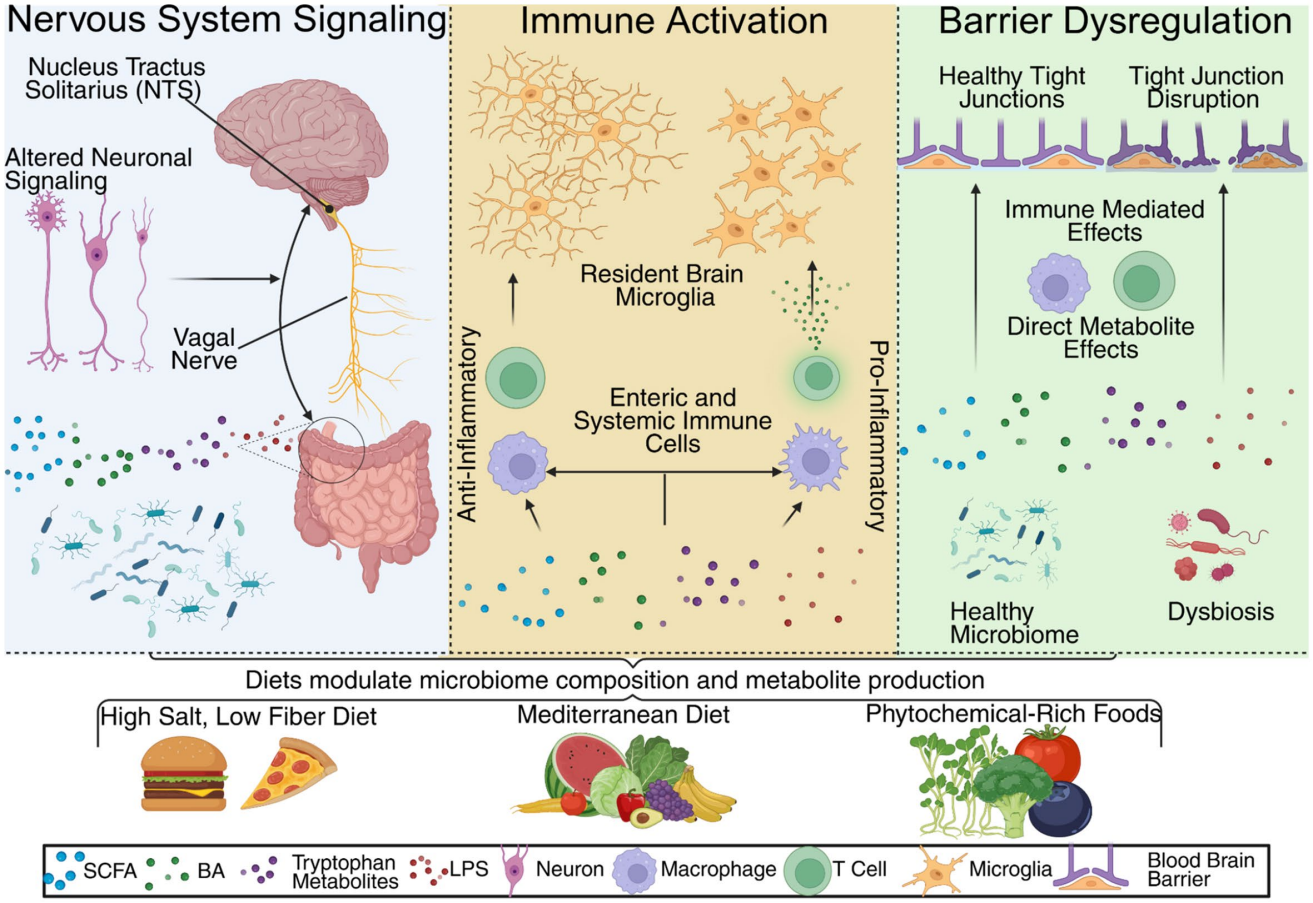
Mechanisms of how diet influences the gut brain axis. Depicting three mechanisms of the gut brain axis of importance for dietary interventions. Different types of diets can modulate the microbiome to produce different metabolites. These metabolites can affect nervous system signaling through the ENS and vagal nerve. Immune activation via these metabolites either enterically or systemically can lead to activation of resident brain microglia. Dietary metabolites have also been shown to either protect blood brain barrier tight junctions or disrupt them, both through direct effects and immune mediated effects. Created in BioRender. Glenn, E. (2026) https://BioRender.com/r99x90a.

Dietary inputs shape the GBA (Figure 1; Tables 2, 3) primarily through their effects on gut microbial composition and metabolic output, thereby influencing immune tone, barrier integrity, and neurochemical signaling (Bonaz et al., 2018; Ma et al., 2019; Ojeda et al., 2021; Bostick et al., 2022; Taraskina et al., 2022; Yuan et al., 2023; Loh et al., 2024; Warren et al., 2024; Yang et al., 2024). Diet-driven effects on brain health emerge from coordinated changes in microbial community structure, metabolite availability, and host-microbe signaling pathways, rather than from single nutrients or isolated taxa (Kliewer and Mangelsdorf, 2015; Coretti et al., 2024; Jaberi et al., 2024).

### 2.1 Microbiota-derived bioactive metabolites

Diet-microbiota interactions generate a diverse array of bioactive microbial metabolites that serve as key molecular intermediaries linking dietary inputs to GBA signaling (Son et al., 2025). Through the fermentation and biotransformation of dietary fibers, amino acids, and phytochemicals, the gut microbiota produces metabolites that can engage neural, immune, endocrine, and barrier pathways relevant to neuroinflammation and neurodegeneration (Ashique et al., 2024). These metabolites influence CNS function both indirectly, by modulating gut barrier integrity, immune activation, and vagal signaling, and directly, by entering systemic circulation and interacting with receptors expressed at the BBB and within neural tissue (Son et al., 2025).

Emerging research identifies specific microbial metabolites as both mediators of neurodegeneration and potential therapeutic targets (Kliewer and Mangelsdorf, 2015). Metabolites such as butyrate and indole derivatives exhibit anti-inflammatory and antioxidant properties, although their effects may vary depending on context, dose, and disease stage (Kliewer and Mangelsdorf, 2015). Inconsistencies across studies reflect the complexity of microbiota-brain interactions and arise from differences in study design, populations, and microbial profiling methods. Nevertheless, converging evidence supports a model in which gut community instability alters metabolite availability, thereby influencing neuroimmune function, glial activation, BBB integrity, and amyloid pathology (Kliewer and Mangelsdorf, 2015). Functional modeling in longitudinal MS cohorts further underscores the clinical relevance of these pathways, demonstrating that microbiota profiles associated with disease progression are depleted in SCFA production and enriched for oxidative metabolic pathways (Montgomery et al., 2024a,b).

#### 2.1.1 Short-chain fatty acids

Microbial metabolites produced through dietary fiber fermentation, especially SCFAs like butyrate, propionate, and acetate, act as central regulators of barrier integrity and immune tone along the GBA (Parker et al., 2019). In the intestine, butyrate serves as a primary energy substrate for colonocytes and supports epithelial barrier maintenance by stabilizing hypoxia-associated signaling and promoting mucus production and tight junction assembly (Kelly et al., 2015). SCFAs additionally reinforce epithelial integrity through FFAR2/FFAR3 signaling and histone deacetylase (HDAC) inhibition, which suppress NF-kB-driven inflammatory pathways and enhance tight junction protein expression (Parada Venegas et al., 2019). *In vitro* epithelial models further demonstrate that butyrate directly upregulates claudin-1, occludin, and ZO-1 via activation of the Akt signaling pathway, providing mechanistic evidence that SCFAs strengthen epithelial architecture at the cellular level (Yan and Ajuwon, 2017). Together, these pathways are thought to limit intestinal permeability and systemic immune activation, thereby potentially reducing the translocation of pro-inflammatory signals that can propagate neuroinflammation.

Beyond the gut, SCFAs influence distal neuroimmune compartments relevant to neurodegeneration. Germ-free mice exhibit increased BBB permeability and reduced endothelial tight junction proteins, defects that are reversed by microbial colonization or SCFA supplementation (Braniste et al., 2014). In parallel, microbiota-derived SCFAs are implicated in supporting microglial maturation and immune homeostasis, as germ-free and antibiotic-treated mice display immature microglial phenotypes, altered gene expression, and impaired innate immune responses (Erny et al., 2015). Restoration of a complex microbiota or direct SCFA supplementation partially rescues these defects, while deletion of the SCFA receptor FFAR2 recapitulates microglial abnormalities observed under germ-free conditions, highlighting a critical role for SCFA-mediated peripheral immune signaling (Erny et al., 2015). Collectively, these findings position SCFAs as key molecular intermediaries that may link diet-microbiota interactions to barrier integrity, immune regulation, and neuroprotective capacity across the GBA, although these mechanisms are primarily supported by *in vitro* and animal studies, and their translational relevance in humans remains to be fully established.

Additional preclinical studies further support these observations, demonstrating that SCFA supplementation or microbial colonization can partially restore microglial development and function in germ-free models, with compounds such as acetate and butyrate associated with shifts toward more homeostatic and anti-inflammatory microglial phenotypes, including reduced expression of pro-inflammatory cytokines (e.g., TNF-α, IL-1β) (Abdel-Haq et al., 2019; Suganya and Koo, 2020). Butyrate acts primarily through histone deacetylase (HDAC) inhibition to regulate microglial gene expression, while acetate signals through FFAR2, to modulate inflammatory signaling cascades and cytokine production (Ashique et al., 2024). Through these complementary mechanisms, gut microbiota-derived SCFAs function as key molecular signals that couple microbial metabolic activity to microglial maturation, immune homeostasis, and neuroprotective capacity (Abdel-Haq et al., 2019). Dysregulation of this interface has been associated with increased neurotoxic cytokine production and oxidative stress, contributing to the pathophysiology of AD and PD (Yadav et al., 2022). Consequently, low-fiber Western-style dietary patterns that reduce SCFA availability may shift GBA signaling toward increased permeability, immune activation, and vulnerability to neurodegenerative processes (Yadav et al., 2022).

#### 2.1.2 Tryptophan metabolites and serotonin-related signaling

Gut microbial metabolism of dietary tryptophan generates a range of bioactive compounds, including indoles, cresols, and imidazole derivatives, that play important roles in neuroimmune regulation (Ashique et al., 2024). Microbial regulation of tryptophan metabolism also influences host serotonin production. Approximately 90% of the body’s serotonin (5-hydroxytryptamine, 5-HT) is produced in the GI tract, where its biosynthesis is tightly regulated by the gut microbiota (Yano et al., 2015). Microbial regulation of tryptophan metabolism, specifically through modulation of tryptophan hydroxylase 1 (TPH1) expression in enterochromaffin (EC) cells, represents a key pathway by which microbes influence host serotonin production (Gershon and Margolis, 2021). Preclinical studies indicate that spore-forming bacteria, including clostridial taxa, can promote TPH1 expression and serotonin release via luminal metabolites like deoxycholate, α-tocopherol, and tyramine (Gershon and Margolis, 2021). Some microbial species have also been shown, primarily in experimental models, to synthesize serotonin directly, independent of host TPH1 (Gershon and Margolis, 2021). For example, a consortium containing Limosilactobacillus mucosae and *Ligilactobacillus ruminis* converts 5-hydroxytryptophan to serotonin, increasing luminal serotonin levels and improving colonic innervation and motility in germ-free Tph1-deficient mice (Moretti et al., 2025). While these findings provide mechanistic insight, their relevance to human physiology remains to be fully established. Although peripheral serotonin does not readily cross the BBB, it can influence CNS function indirectly through vagal afferents, immune signaling, and ENS activity (Moretti et al., 2025).

Indole derivatives can activate the AhR, leading to suppression of neuroinflammatory signaling in the CNS (Yang et al., 2024). However, AhR signaling is highly ligand- and context-dependent, and distinct microbial tryptophan metabolites can either suppress or exacerbate neuroinflammatory pathways depending on molecular structure, cellular target, and disease state. Through this pathway, indoles influence glial phenotypes and may bias microglial responses toward less inflammatory states (Coretti et al., 2024). In parallel, select indole metabolites, including 3IS, modulate gut-brain communication through neural pathways (Sittipo et al., 2022). Specifically, 3IS elicits prolonged activation of vagal afferents via transient receptor potential ankyrin 1 (TRPA1)-mediated signaling, likely mediated through enteroendocrine cells instead of acting directly on the vagal neurons (Jameson et al., 2025; Ma et al., 2019). Together, these mechanisms link microbial tryptophan metabolism to central autonomic and immune regulation and highlight how dietary tryptophan quality, along with polyphenols and fiber that favor indole-producing taxa, can shape GBA signaling (Coretti et al., 2024).

Montgomery and colleagues provided a clear example of how microbial tryptophan metabolism can exacerbate CNS autoimmunity in a diet-dependent manner (Montgomery et al., 2022). Using the EAE model, *Lactobacillus reuteri* were enriched for tryptophan-catabolizing enzymes, enabling the conversion of host dietary tryptophan into multiple bioactive metabolites, including indoles, cresols, and imidazole derivatives (Montgomery et al., 2022). Mice colonized with *L. reuteri* developed more severe disease when maintained on a high-tryptophan diet, accompanied by expansion of IL-17–producing γδ T cells, whereas dietary tryptophan restriction attenuated both the microbial metabolite signature and disease severity (Montgomery et al., 2022). Mechanistic analyses further demonstrated that several *L. reuteri*-derived indoles and imidazoles act as AhR ligands that enhance IL-17 production, illustrating how microbial processing of dietary tryptophan can bias adaptive immune responses toward a pathogenic Th17/γδ17 phenotype and worsen neuroinflammatory outcomes (Montgomery et al., 2022). Importantly, this contrasts with the anti-inflammatory effects of other indole metabolites and reflects the context-dependent nature of AhR signaling, in which ligand structure, cellular target, and disease state determine whether AhR activation suppresses or exacerbates neuroinflammation (Montgomery et al., 2022).

#### 2.1.3 Bile acid metabolites

BAs are microbiota-regulated metabolites that function as important signaling molecules within the GBA (Sittipo et al., 2022). Primary BAs synthesized in the liver are modified by gut microbes into secondary BAs, thereby shaping the circulating BAs pool and its signaling properties (Montgomery et al., 2024a). Within the intestine, BAs activate gut-brain communication primarily through TGR5, which is expressed on enteroendocrine cells and vagal afferent terminals and indirectly couples luminal BAs signals to neural and hormonal outputs (Jameson et al., 2025; Ma et al., 2019). Activation of intestinal TGR5 promotes the release of hormones such as glucagon-like peptide 1 (GLP-1) and can indirectly engage vagal afferent signaling to brainstem and hypothalamic nuclei involved in metabolic and neuroimmune regulation (Montgomery et al., 2024a).

Beyond vagal-mediated pathways, BAs also act as systemic signaling molecules capable of influencing CNS function directly (Ferrell and Chiang, 2021). Although the majority of BAs undergo enterohepatic recycling, a small fraction enters systemic circulation and can access the brain via proposed mechanisms such as passive diffusion or transporter-mediated mechanisms at the BBB (Ferrell and Chiang, 2021). Receptors for BAs, including farnesoid X receptor (FXR) and TGR5, are expressed in neurons, astrocytes, and microglia, where BAs signaling has been shown, primarily in preclinical models, to modulate inflammation, metabolic pathways, and cellular stress responses (Ferrell and Chiang, 2021). Consistent with this, BAs have been detected in cerebrospinal fluid, and altered BAs composition has been associated with neurodegenerative conditions such as AD and PD (Ferrell and Chiang, 2021). Importantly, BAs signaling exerts highly context-dependent and sometimes opposing effects in the CNS. Certain secondary BAs activate TGR5 to promote anti-inflammatory and neuroprotective responses, whereas others may increase BBB permeability and neuroinflammation, although these effects vary depending on BA species, receptor engagement, and experimental model, potentially through FXR- or sphingosine-1-phosphate receptor 2 (S1PR2)–dependent mechanisms (Ferrell and Chiang, 2021). These divergent effects highlight the importance of BAs composition, receptor specificity, and tissue context, and underscore that the net impact of BA signaling on neurological outcomes remains incompletely understood, particularly in human studies (Ferrell and Chiang, 2021).

In parallel, intestinal BAs signaling also engages endocrine pathways that indirectly influence brain function. Activation of intestinal FXR induces fibroblast growth factor 19 (FGF19) release, while TGR5 activation on enteroendocrine L-cells stimulates GLP-1 secretion (Mertens et al., 2017). Both hormones can signal to the brain via the circulation and vagal afferent pathways, particularly targeting hypothalamic and brainstem circuits that regulate energy balance, glucose homeostasis, and neuroendocrine function (Kliewer and Mangelsdorf, 2015; Mertens et al., 2017). Together, these mechanisms position BAs as a key mechanistic link between diet-microbiota interactions and central neuroimmune and metabolic regulation within the GBA.

#### 2.1.4 Polyphenol- and phytochemical-derived microbial metabolites

In addition to primary microbial metabolites, the gut microbiota also generates a wide range of bioactive metabolites that influence neuroinflammatory and neurodegenerative processes (Rowland et al., 2018). These compounds are not primary microbial metabolites, but rather secondary products of microbial biotransformation of dietary phytochemicals, particularly polyphenols. For example, ellagitannins are converted by gut microbes into urolithins, particularly urolithin A, which has demonstrated anti-inflammatory and neuroprotective effects by activating mitophagy and modulating microglial function (Rowland et al., 2018). Flavonoids like quercetin and rutin are metabolized by gut microbes into phenylacetic acids and dihydroxybenzoic acids, which can cross the BBB and exert antioxidant and anti-inflammatory effects in the brain (Rowland et al., 2018). Anthocyanins are metabolized by gut microbes into protocatechuic acid, a phenolic compound known to attenuate neuroinflammation and oxidative stress in preclinical *in vivo* and *in vitro* experimental models of neurodegeneration diseases, including AD, PD, and MS (Fujisaka et al., 2023).

#### 2.1.5 Microbiota-responsive hormonal mediators

Hormones such as ghrelin, leptin, and GLP-1 are key mediators in GBA communication that are dynamically regulated by both dietary inputs and microbial activity. Although these hormones are synthesized by host tissues, microbial metabolism influences their secretion, receptor sensitivity, and downstream signaling pathways (Bonfili et al., 2017). Ghrelin plays a role in appetite regulation and exerts neuroprotective effects by promoting hippocampal plasticity and modulating neuroinflammation (Bonfili et al., 2017). Circulating ghrelin levels decline with aging and cognitive impairment, but can be partially restored through microbiota-targeted probiotic interventions, such as SLAB51, which have been associated with improvements in memory performance in preclinical models (Bonfili et al., 2017). Leptin, an adipose-derived hormone, regulates synaptic function and neuroinflammation and is reduced in obesity-associated cognitive decline. Impaired leptin signaling (leptin resistance), rather than absolute leptin deficiency, is commonly observed in obesity-associated cognitive decline (Bonfili et al., 2017). Dietary patterns associated with improved metabolic health, such as fiber-rich, plant-based diets that reduce adiposity and systemic inflammation, have been shown to enhance leptin sensitivity, which in turn has been linked to reductions in amyloid-*β* pathology and improved neuronal survival (Bonfili et al., 2017). GLP-1, an incretin hormone, exhibits strong neuroprotective and anti-inflammatory effects, and its signaling can be enhanced through microbiota-targeted therapies, offering potential benefits in AD and PD treatment (Bonfili et al., 2017). Microbiota-dependent increases in GLP-1 secretion, particularly through fermentation of dietary fibers into SCFAs, enhance GLP-1 signaling and may contribute to improved cognitive and metabolic outcomes in AD and PD (Jaberi et al., 2024).

Diet has a strong effect on hormone-driven gut-brain communication by influencing both microbial metabolism and host endocrine responses (Gogga et al., 2022). Caloric restriction and high-fiber dietary patterns consistently increase GLP-1 secretion, while dietary macronutrient composition influences ghrelin dynamics, with higher protein and fat intake generally suppressing postprandial ghrelin levels, and low-energy or fasting states promoting ghrelin release (Gogga et al., 2022). Thus, gut hormones are synthesized by the host but are dynamically regulated by dietary inputs and microbial activity, positioning them as important intermediaries linking diet, the microbiota and brain function (Gogga et al., 2022).

### 2.2 Microbiota-derived neurotransmitters and neuroactive compounds

In addition to producing bioactive metabolites, the gut microbiota influences GBA signaling through the production and modulation of neuroactive compounds involved in host neurotransmission (Loh et al., 2024). Although most neuroactive compounds are synthesized by host cells, microbial metabolism strongly influences their availability, precursor flux, receptor engagement, and downstream signaling, thereby shaping CNS function and disease risk (Loh et al., 2024).

#### 2.2.1 Dopamine

Dopamine is essential for motor control, motivation, and reward processing and is centrally implicated in PD through degeneration of nigrostriatal dopaminergic neurons (Loh et al., 2024). While CNS dopamine is host-derived, the gut microbiota influences dopaminergic signaling indirectly by regulating precursor availability (tyrosine and L-DOPA) and peripheral catecholamine metabolism (Hamamah et al., 2022). Certain *Lactobacillus* and *Bifidobacterium* species can metabolize tyrosine into dopamine-like compounds, though their direct impact on CNS dopamine remains unclear (Hamamah et al., 2022). Microbial dysbiosis and loss of barrier-supportive metabolites may disrupt gut-brain dopamine signaling and influence L-DOPA absorption and peripheral conversion, thereby affecting responsiveness in PD (Daubner et al., 2011; Peipert et al., 2025). Dietary protein quality and microbial composition jointly shape this pathway by determining precursor availability and drug metabolism (Daubner et al., 2011).

#### 2.2.2 Acetylcholine

Acetylcholine plays a critical role in cognitive processes, particularly learning, memory, and attention (Picciotto et al., 2012). Cholinergic dysfunction is a hallmark of AD and contributes to deficits in learning and memory (Parker et al., 2019). Gut microbiota disruption has been shown to increase acetylcholinesterase (AChE) activity, reducing acetylcholine availability and impairing cholinergic signaling (Picciotto et al., 2012). Some gut microbes, including *Lactobacillus plantarum*, can synthesize acetylcholine or influence its metabolism indirectly (Picciotto et al., 2012). In preclinical AD models, interventions using *L. plantarum* and prebiotics like fructooligosaccharides (FOS) have restored acetylcholine levels, reduced acetylcholinesterase activity, and improved cognitive outcomes (Qu et al., 2024). Dietary prebiotics like non-digestible fibers (FOS, inulin), help to foster microbial communities that can preserve acetylcholine signaling. At the same time, polyphenols can inhibit AChE activity which strengthens diet’s role in maintaining cholinergic tone (Divyashri et al., 2021). Thus, although acetylcholine is synthesized by host neurons, microbial regulation of AChE activity and community composition plays a significant role in maintaining cholinergic tone.

#### 2.2.3 Glutamate

Glutamate is the principal excitatory neurotransmitter in the CNS and is vital for synaptic plasticity and memory formation (Gruenbaum et al., 2024). However, excessive glutamate activity can lead to excitotoxicity, which is implicated in the pathology of AD and PD (Gruenbaum et al., 2024). The gut microbiota influences glutamatergic balance by regulating glutamine availability and amino acid metabolism. Dysbiosis has been associated with altered glutamate signaling and increased excitatory tone, particularly in PD (Warren et al., 2024). Probiotic supplementation with strains that regulate glutamine-glutamate balance has shown promise in reducing excitotoxicity and improving behavioral outcomes in preclinical models (Gruenbaum et al., 2024). Dietary protein intake helps to determine glutamine-glutamate flux, while in comparison, diets that are high in fat, sugar, salt and low in fiber, characteristic of Western-style dietary patterns, may increase excitotoxic risk by promoting microbial community instability and reducing the availability of protective metabolites (Ma et al., 2018; Wu et al., 2022). In contrast, balanced, fiber-rich plant-based diets support microbial taxa that help regulate glutamine-glutamate cycling. Although glutamate signaling in the CNS is host-derived, it is strongly influenced by microbial regulation of precursor availability, shaping excitatory tone and vulnerability to excitotoxic injury (Ma et al., 2018; Wu et al., 2022).

#### 2.2.4 Gamma-aminobutyric acid

GABA is the major inhibitory neurotransmitter in the CNS, and its dysregulation has been implicated in anxiety, depression, and neurodegenerative disorders (Miri et al., 2023). While GABA acts primarily as a host neurotransmitter, certain gut bacteria such as *Lactobacillus brevis* and *Bifidobacterium dentium* can synthesize GABA from glutamate, thereby contributing to the gut GABA pool and modulating enteric and central neuronal activity (Miri et al., 2023). Microbially derived GABA may influence CNS function indirectly through vagus nerve signaling and by shaping systemic inflammatory tone (Yan and Ajuwon, 2017). Several studies have shown that GABA-producing probiotics improve mood, reduce stress responses, and enhance cognitive function in both animal models and human clinical trials (Braga et al., 2024; Casertano et al., 2024; Patterson et al., 2019). Dietary fiber and fermented foods help to enrich GABA-producing taxa, while diets that are high in processed fats and sugars may suppress their abundance which leads to a reduction in GABA availability and its inhibitory effects on CNS excitability (Hills et al., 2019). Together, these findings highlight that plant-derived phytochemicals exert their neuroprotective effects through heterogeneous, microbiota-dependent pathways that vary widely in bioavailability, metabolic fate, and signaling targets (Qu et al., 2024).

## 3 Dietary bioactives from cruciferous vegetables and GBA modulation

Among dietary bioactives, compounds derived from cruciferous vegetables, including broccoli sprouts and Brussels sprouts, provide a well-defined and mechanistically tractable model for studying diet-microbiota-brain interactions, as their glucosinolate-to-isothiocyanate conversion requires enzymatic and microbial activity and converges on conserved antioxidant and anti-inflammatory response pathways (Bessetti and Litwa, 2025). This coupling has been demonstrated across *in vitro* systems, animal models, and early-stage human studies, including work from our research group and others, making cruciferous bioactives a useful example for interrogating diet-microbiota-brain interactions (Holcomb et al., 2023; Holman et al., 2023; Alaba et al., 2024). Cruciferous vegetables provide abundant glucosinolates that certain microbes and plant myrosinase convert to isothiocyanates such as sulforaphane (SFN), a bioactive with strong anti-inflammatory and antioxidant activity (Subedi et al., 2019). In mechanistic neuroinflammation models, SFN suppresses MAPK (notably JNK) signaling and downstream NF-kB/AP-1 activity in LPS-activated microglia, while inducing Nrf2/HO-1 and increasing anti-inflammatory cytokines (Subedi et al., 2019). Broccoli sprouts are particularly rich in glucoraphanin, the precursor to SFN, which can be efficiently converted to its bioactive form via plant and/or microbial myrosinase activity. SFN is bioavailable, can access the CNS, and has been linked to neuroprotection through antioxidant and anti-inflammatory pathways (Subedi et al., 2019). The following subsections summarize *in vitro, in vivo*, and human studies examining how SFN influences microglial activation, oxidative stress, barrier integrity, and neurodegenerative pathology.

### 3.1 *In vitro* evidence of SFN on neuroinflammation

SFN exerts potent anti-inflammatory and antioxidant effects by modulating key signaling pathways in microglial cells (Subedi et al., 2019; Feng et al., 2023). In LPS-stimulated murine BV2 microglial cells, SFN significantly inhibited MAPK pathway components, including c-Jun N-terminal kinase (JNK), p38, and ERK, leading to a decrease in pro-inflammatory cytokines like TNF-*α*, IL-6, and IL-1*β*. SFN also downregulated iNOS, COX-2, NO, and PGE2 production while enhancing anti-inflammatory cytokines IL-10 and IL-4 via the Nrf2/heme oxygenase 1(HO-1) axis (Subedi et al., 2019). This reduction in pro-inflammatory cytokine expression was mechanistically linked to SFN-mediated suppression of NF-kB and AP-1 transcriptional activity, key regulators of inflammatory gene expression (Subedi et al., 2019). Notably, SFN was more effective in reducing inflammation and promoting cell survival than allyl isothiocyanate (AITC), another Brassicaceae-derived compound, for anti-inflammatory and pro-survival effects in microglia (Subedi et al., 2019; Feng et al., 2023).

In a study done by Han and colleagues, SFN demonstrated neuroprotective effects by promoting neural stem cell (NSC) proliferation and differentiation at low concentrations (<10 μM) via the Wnt signaling pathway (Han et al., 2017). SFN enhances NSC viability, increases neurosphere formation, and stimulates neuronal differentiation which is evidenced by elevated Ki-67 and Tuj-1 expression levels (Han et al., 2017). The Wnt signaling pathway is critical to these effects, with SFN upregulating key proteins such as β-catenin and cyclin D1 (Han et al., 2017). Inhibition of this pathway via DKK-1 reduces SFN’s beneficial effects which demonstrates its mechanistic role (Han et al., 2017). Notably, SFN exhibited dose-dependent effects, with concentrations above 10 μM becoming cytotoxic, underscoring the importance of physiologically relevant dosing strategies (Han et al., 2017). Beyond its established antioxidant and anti-inflammatory actions via Nrf2 activation, SFN’s ability to modulate Wnt signaling makes it a potentially promising option for treating neurodegenerative conditions marked by oxidative stress, inflammation, and reduced neurogenesis (Han et al., 2017). Further research is required to validate these findings in clinical settings and optimize therapeutic applications (Han et al., 2017).

Activation of Nrf2 by SFN is closely tied to its ability to promote cytoprotection, maintain mitochondrial integrity, and support microglial phenotype switching (Eren et al., 2018). In murine N9 microglial cells, SFN mitigated LPS-induced inflammation, oxidative stress, and cell death through the ERK1/2-Nrf2 pathway (Eren et al., 2018). Nrf2 activation led to increased expression of antioxidant genes like HO-1, Gclc, and Srxn1, while suppressing inflammatory cytokines and miR-155. SFN inhibited NF-κB and AP-1, reduced reactive oxygen and nitrogen species, and preserved mitochondrial health (Eren et al., 2018). This prevented apoptosis and necrosis. SFN promoted a shift away from pro-inflammatory microglial activation toward a stress-responsive, oxidative stress-adaptive microglial (Mox-like) phenotype (Eren et al., 2018). In co-culture systems, SFN-pretreated N9 murine microglia reduced microglia-mediated neurotoxicity toward SH-SY5Y neuronal cells and improved neurite outgrowth, further underscoring its therapeutic potential in neuroinflammatory contexts (Eren et al., 2018).

Beyond acute anti-inflammatory and antioxidant actions, SFN can influence epigenetic regulation of gene expression (e.g., inhibiting HDACs and DNA methyltransferases), which may reprogram transcriptional networks involved in proteostasis, stress resilience, and cellular aging (Santín-Márquez et al., 2019; Subedi et al., 2019). For example, SFN shows disease-specific promise in AD by epigenetically enhancing Nrf2 signaling (Zhao et al., 2018). In a neuronal AD-like cell model (mouse neuroblastoma N2a cells stably expressing human Swedish mutant APP; N2a/APPswe), SFN decreased DNA methylation at the Nrf2 promoter, thereby increasing Nrf2 expression and nuclear translocation (Zhao et al., 2018). Downstream of the Nrf2 activation, SFN increased HO-1 and NQO1 expression, reduced ROS and MDA, and increased superoxide dismutase (SOD) activity (Casertano et al., 2024). These cytoprotective shifts were accompanied by reduced inflammatory signaling outputs, including decreased NF-κB-associated IL-1β, IL-6, COX-2, and iNOS expression (Casertano et al., 2024). SFN also reduced Aβ peptide levels central to AD pathology (Casertano et al., 2024). These findings highlight that SFN exhibits antioxidative and anti-inflammatory effects, suggesting its potential as a therapeutic agent for AD. However, further *in vivo* studies are needed to confirm its role and efficacy in AD treatment (Zhao et al., 2018).

### 3.2 *In vivo* animal studies

In vivo evidence for SFN-mediated neuroprotection has been demonstrated in rodent models of focal cerebral ischemia (Zhao et al., 2006). In adult male Long-Evans rats subjected to transient focal ischemia via common carotid artery/middle cerebral artery (CCA/MCA) occlusion for 3 h followed by reperfusion, intraperitoneal administration of SFN (95 mg/kg) 15 min after ischemia onset significantly reduced infarct volume, as assessed by 2,3,5-triphenyltetrazolium chloride (TTC) staining (Zhao et al., 2006). This model induces cortical infarction through mechanical arterial occlusion, mimicking ischemic stroke with subsequent reperfusion injury. Systemic SFN treatment increased cortical expression of the Nrf2-responsive gene HO-1, indicating central activation of antioxidant response element (ARE)-dependent cytoprotective pathways. Although direct BBB permeability was not quantified, induction of HO-1 within brain tissue supports brain bioavailability following systemic delivery (Zhao et al., 2006). SFN has also been shown to protect against Amyotrophic Lateral Sclerosis (ALS) by preserving motor neurons through activation of the Nrf2/ARE pathway, with enhanced efficacy observed when combined with riluzole (Subedi et al., 2019).

Neuroprotection has also been demonstrated in a neonatal hypoxic–ischemic encephalopathy (HIE) model (Kapoor et al., 2022). In postnatal day 7 Sprague–Dawley rat pups subjected to the modified Rice-Vannucci model (unilateral common carotid artery ligation followed by 90 min of normobaric hypoxia at 8% oxygen), SFN pretreatment (5 mg/kg, i.p.) administered 24 h prior to injury improved hippocampal glucose metabolism at 24 h and 1 week post-insult as measured by ^18F-FDG μCT/PET imaging (Kapoor et al., 2022). Morphometric analysis 5 weeks post-injury showed a trend toward preservation of hippocampal thickness in SFN-treated animals compared to untreated HIE controls, although motor outcomes assessed by ladder rung walking were not improved (Kapoor et al., 2022). These findings suggest that SFN modulates metabolic vulnerability in the immature hippocampus following hypoxic–ischemic injury, with region-specific effects that appear more pronounced in hippocampal than cortical tissue (Kapoor et al., 2022).

Kamal et al. provided a comprehensive review of previously published in vivo rodent models (e.g., MOG35-55-induced EAE using C57BL mice, MCAO stroke using a rat model, MPTP-induced Parkinson’s disease using C57BL/6 mice, transgenic AD mice, SOD1G93A transgenic ALS rodent model) and complementary *in vitro* systems demonstrating the neuroprotective effects of SFN (Kamal et al., 2022). In EAE, SFN reduced disease severity by approximately 40%, delayed symptom onset, and improved clinical scores (Kamal et al., 2022). This was mediated primarily through suppression of inflammatory infiltrates and demyelination in the spinal cord (Kamal et al., 2022). Its neuroprotective mechanism in MS involved downregulation of JNK/ERK1/2/NF-κB signaling, enhancement of regulatory T-cell responses, reduction of oxidative stress, and preservation of BBB integrity via restoration of claudin and ZO-1 expression (Kamal et al., 2022). In prion disease models, which typically involve exposure of neuronal cells to neurotoxic prion protein fragments to mimic protein misfolding and aggregation seen in prion disorders, SFN mitigated neurotoxicity by inducing autophagy through activation of the AMPK pathway (Kamal et al., 2022). SFN was also found to protect against schizophrenia-related oxidative stress by preventing dopamine-induced lipid peroxidation, increasing glutathione levels, and enhancing antioxidant enzyme activity (Kamal et al., 2022). It also mitigated epilepsy-associated oxidative stress and cognitive impairment by stimulating the Nrf2/ARE pathway and reduced depressive symptoms through modulation of the hypothalamic–pituitary–adrenal axis and inflammatory response (Kamal et al., 2022). These findings show SFN’s neuroprotective potential, particularly in AD, PD, ischemic stroke, ALS, MS, prion diseases, epilepsy, schizophrenia, and depression (Kamal et al., 2022). These were largely mediated through SFN’s ability to regulate oxidative stress, inflammation, and neuronal survival pathways (Kamal et al., 2022).

The study done by He and colleagues investigates the role of Nrf2 in regulating TREM2 and its implications for depression-like behaviors in male adult C57BL mice (8 weeks old) with CD1 male mice (14 weeks old) as aggressors (He et al., 2022). Nrf2 is a key regulator of antioxidant and anti-inflammatory responses and it initiates TREM2 transcription which promotes the expression of anti-inflammatory microglial arginase 1 + phenotype in the medial prefrontal cortex (mPFC) (He et al., 2022). Activation of Nrf2 via SFN was found to enhance TREM2 expression and ameliorated depression-like behaviors in a chronic social defeat stress (CSDS) mouse model (He et al., 2022). Knockout of Nrf2 or downregulation of TREM2 diminished the arginase 1 + microglial phenotype and exacerbated depressive behaviors which linked the pathway to the brain-derived neurotrophic factor (BDNF)-tropomyosin receptor kinase B (TrkB) signaling cascade (He et al., 2022). This research highlights that Nrf2 activation by SFN can induce an anti-inflammatory microglial phenotype, increase BDNF levels, and reverse reduced dendritic spine density in the mPFC of CSDS mice (He et al., 2022). TREM2 was shown to influence Nrf2 expression, suggesting a positive feedback loop (He et al., 2022). Downregulation of TREM2 through heteroduplex oligonucleotide treatment negated the beneficial effects of Nrf2 activation on depression-like behaviors which emphasized the role that TREM2 has in this pathway (He et al., 2022). The study demonstrated the therapeutic potential of targeting the Nrf2-TREM2 axis in modulating microglial function and improving depressive symptoms, with implications for understanding the neuroinflammatory basis of depression (He et al., 2022). However, further research is needed to fully understand the mechanistic links between TREM2, microglial BDNF production, and its contribution to depression pathogenesis (He et al., 2022). Similarly, Tang et al. demonstrated SFN’s potential for alleviating depression-like symptoms by upregulating BDNF transcription and suppressing MeCP2 in microglia, thereby supporting dendritic spine repair and synaptic resilience (Tang et al., 2022). Both studies underscore SFN’s promise as an intervention that targets microglial dysfunction, oxidative stress, and inflammation in the pathogenesis of mood disorders (He et al., 2022; Tang et al., 2022). Together, these findings suggest a bidirectional relationship in which SFN modulates microglial and neural function, while microbial composition and enzymatic capacity determine SFN bioactivation and bioavailability (Han et al., 2017; Divyashri et al., 2021; He et al., 2022; Kamal et al., 2022; Tang et al., 2022).

### 3.3 Human studies

Human evidence for SFN in neurodegeneration is emerging, with early clinical studies and pilot trials reporting signals of benefit across cognitive, behavioral, and systemic oxidative/inflammatory outcomes (Schepici et al., 2020). Reported benefits include improved cognitive or behavioral measures and reductions in systemic oxidative/inflammatory biomarkers in small trials (Schepici et al., 2020). The lipophilic nature of SFN ensures high bioavailability which makes it a promising addition to current therapies for neurodegenerative diseases (Schepici et al., 2020). While its efficacy and low neurotoxicity supports SFN as a potential therapeutic agent, further studies are necessary to fully understand its mechanisms and optimize its clinical application (Schepici et al., 2020).

An ongoing randomized controlled trial by Liu and colleagues is currently investigating whether SFN can improve cognitive function in patients with frontal brain damage (Liu et al., 2020). Cognitive deficits resulting from conditions such as trauma, tumors, and cerebrovascular diseases significantly impair quality of life and clinical outcomes (Liu et al., 2020). In this study, 90 participants will be randomly assigned to receive either SFN or placebo for 12 weeks (Liu et al., 2020). Cognitive assessments, neuroimaging (T1-weighted and resting-state functional MRI), and biochemical analyses (measuring BDNF, GSH, Glu, and GABA levels in the brain and blood) will be conducted both at baseline and after the 12-week period (Liu et al., 2020). The primary outcome is improved performance on cognitive tests, with secondary outcomes including shifts in neuropsychiatric measures, brain metabolites, and gut microbiota composition (Liu et al., 2020). Although results are not yet available, the study design enables evaluation of whether changes in neuroimaging, biochemical markers, and gut microbiota profiles correlate with cognitive outcomes (Liu et al., 2020). By exploring sulforaphane’s antioxidant and neuroprotective mechanisms, the study aims to evaluate its potential as an adjunct therapy for enhancing cognitive function and mitigating the long-term effects of brain trauma, tumors, and cerebrovascular damage (Liu et al., 2020). Notably, this research integrates gut microbiota profiling with neuroimaging and biochemical data, which may provide insight into mechanistic pathways if findings are confirmed (Liu et al., 2020).

Beyond clinical testing, SFN is widely discussed as a translational nutraceutical because it engages Nrf2-mediated cytoprotective programs rather than targeting a single downstream symptom pathway (Houghton, 2019). Unlike many conventional pharmaceuticals that focus on alleviating symptoms, SFN targets upstream cellular defense mechanisms through the activation of the transcription factor Nrf2 (Houghton, 2019). This pathway plays a key role in regulating antioxidant and detoxification responses (Houghton, 2019). Compared to other phytochemicals, SFN has relatively high bioavailability, which enhances its potential to exert biological effects in humans (Houghton, 2019). Activation of Nrf2 by SFN increases the expression of cytoprotective enzymes, thereby helping to reduce oxidative stress and inflammation which are two central processes in the development of diseases (Houghton, 2019). Clinical studies have shown that SFN may benefit individuals with neurodegenerative disorders by supporting redox homeostasis, modulating immune responses, and dampening inflammation (Houghton, 2019). SFN has also been shown to inhibit *Helicobacter pylori*, suggesting additional promise for GI health (Houghton, 2019). Nonetheless, challenges remain, particularly in ensuring consistent potency and bioactivity across SFN-containing supplements (Houghton, 2019). Larger, well-designed clinical trials are still needed to confirm its therapeutic value and determine how SFN might best be integrated into conventional healthcare (Houghton, 2019). While existing human trials remain small and heterogeneous, their consistency across immune, oxidative, and behavioral endpoints underscores the translational potential of SFN-rich foods like broccoli sprouts (Houghton, 2019).

## 4 Possible effects of pharmaceuticals targeting gut inflammation on microbiota and GBA

### 4.1 Off-target effects on the microbiome which improved outcomes

Off-target effects of pharmaceuticals can impact gut microbiota, which can, in turn, impact digestion and availability of microbial byproducts, which can impact neuroinflammation. This link has been most clearly demonstrated for IBD. One meta-analysis found that IBD patients, especially active disease, had increased prevalence for most measures of anxiety or depression (Barberio et al., 2021). Depression and anxiety scores correlate with disease outcome in patients (Duan et al., 2023), and in mouse models (Ghia et al., 2008), and there is evidence that treatment with antidepressants can improve the outcome of colitis in mouse models (Varghese et al., 2006; Ghia et al., 2008).

Anti-inflammatory drugs such as corticosteroids, 5-aminosalicylic acid (5-ASA), and biologics like TNF-*α* inhibitors are commonly used to treat conditions like IBD. The corticosteroid dexamethasone decreased biodiversity of gut microbiota (Huang et al., 2015; Couch et al., 2023)owever; this loss of diversity protected against disease in an IL-10 knockout mouse model, in which mice spontaneously develop a colitis-like condition in response to normal gut microbiota, but after microbiota transplant from dexamethasone-treated mice to IL-10-ko mice, their body weight and gut health markers showed improvement (Huang et al., 2015). *Lactobacillus* bacteria were increased in dexamethasone-treated mice (Warren et al., 2024), which may impact the serotonin pathway due to changes in serum kynurenine (Zhao et al., 2006), as well as through beneficial byproducts. Similarly, 5-ASA treatment changes the gut microbiota populations in mouse models of colitis (Wada et al., 2023) as well as patients (Mehta et al., 2023). After 2 weeks of 5-ASA treatment, the Bacteroidetes phylum showed a decrease in relative abundance, while Actinobacteria and Firmicutes were increased, with the significant increase continuing after 4 weeks for the Actinobacteria phyla (Wada et al., 2023). Using a vertical transmission model for the gut microbiota, a protective effect was observed against DSS-induced colitis in the 5-ASA derived pups at the histological and mRNA levels (Wada et al., 2023).

Notably, in human studies gut microbiota changes seem to be different depending on the patient population and specific biologics. In a pediatric CD population, infliximab, one of the most prescribed TNF-α inhibitors for IBD (Markopoulos et al., 2025), improved gut microbiota diversity (Wang et al., 2018) And contributed to restoring the SCFA-producing bacteria population (Wang et al., 2018). Another study in adult UC patients found that remission of UC after 8 weeks of treatment with adalimumab could be predicted by the relative abundances of 48 gut bacterial strains primarily from the Firmicute phylum (Oh et al., 2024). Vedolizumab, by targeting α_4_β_7_ integrin, a GI-specific adhesion molecule on a subset of T cells (O’Reilly et al., 2023), prevents the trafficking of a subset of T cells to the GI tract, thereby alleviating inflammation in IBD. Similar to anti-TNF therapy, predictors in the gut microbiota have been associated with responsiveness to vedolizumab therapy (Ananthakrishnan et al., 2017). *Burkholderiales* and *Roseburia inulinivorans* taxa were significantly higher in non-remitter baseline samples, and pathway analysis revealed multiple pathways, including the super-pathway of arginine and polyamine biosynthesis, that were significantly different in remitter versus non-remitter baseline samples (Ananthakrishnan et al., 2017).

These pharmaceuticals, by targeting gut inflammation, may have downstream benefits for the brain, potentially through GBA, though they have not been well studied. These agents have the potential to lower neuroinflammatory signaling through modulating immune signaling, microbial metabolites, and/or barrier function. A cross-sectional study found that corticosteroids were linked to a decrease of white matter integrity in the brain (van der Meulen et al., 2022) in a cohort study from the UK. Furthermore, a case control study found that long-term use of prednisone was linked with higher scores in depressive symptom evaluations (Warrington and Bostwick, 2006), and a recent mouse study found this could be mediated via ceramides produced in the gut (Wang et al., 2024). Infliximab has been studied for therapeutic effects in bipolar depression, with potential for acting along the glutamate system to improve clinical outcomes (Mansur et al., 2021). With the current understanding of how the microbiota changes in response to these drug treatments (Wang et al., 2018; Mehta et al., 2023; Wada et al., 2023), it is relevant to have further investigation into how these changes may mediate the effects of these anti-inflammatory drugs on the brain.

### 4.2 Diet to counteract off-target effects on the microbiome

Understanding the bidirectional effects that microbiome and IBD treatment have on each other may allow us to better design microbial intervention that can synergistically affect the efficacy of treatment. Corticosteroids have some evidence of impacting brain function (van der Meulen et al., 2022), and the microbiome is an important predictor of therapeutic success for adalimumab and vedolizumab (Oh et al., 2024; Ananthakrishnan et al., 2017), thus a dietary intervention that leads to a more treatment-responsive microbiome could provide a future pharmaceutical diet dual intervention.

Research has already gone into using diet to supplement pharmacological interventions or alleviate their negative effects (Wei et al., 2025; Esteves et al., 2022). So far, results have been limited in terms of diet-only interventions alleviating the symptoms of IBD patients (Wei et al., 2025). However, there have been some promising results, such as a trial with exclusive enteral nutrition showing an improvement in clinical outcome for CD patients (Guo et al., 2022). Dietary recommendations for patients on glucocorticoids currently involve avoiding ultra-processed foods, intake of calcium of vitamin D, and high protein intake, but further personalizing this diet based on microbiome characteristics could improve the response to side effects of glucocorticoids (Esteves et al., 2022). Similar interventions on antidepressants have been suggested, due to the effects of the microbiome on the GBA (Xu et al., 2023). Putting these results together, dietary interventions remain a strong candidate for improving the responsiveness of different pharmacological treatments for IBD, as well as alleviating the side effects of steroid based therapies.

## 5 Conclusion

Diet-microbiota interactions represent a central and highly modifiable axis through which peripheral metabolic processes influence neuroimmune signaling and brain health. Across neurodegenerative and neuroinflammatory conditions, converging evidence indicates that dietary patterns shape gut microbial composition and metabolic output, regulating gut barrier integrity, immune activation, neurotransmitter signaling, and vagal communication within the GBA (Bonaz et al., 2018; Ma et al., 2019; Ojeda et al., 2021; Bostick et al., 2022; Taraskina et al., 2022;^90,47^; Warren et al., 2024; Yang et al., 2024). These effects emerge from coordinated changes in microbial metabolite profiles, including SCFAs, tryptophan-derived indoles, BAs, and polyphenol-derived compounds, that integrate dietary inputs with host neuroimmune responses, rather than from isolated taxa or single signaling pathways (Kliewer and Mangelsdorf, 2015; Suganya and Koo, 2020; Ojeda et al., 2021; Bostick et al., 2022; Ashique et al., 2024; Coretti et al., 2024; Jaberi et al., 2024).

This review highlights that fiber-rich, plant-based dietary patterns consistently promote microbial functions associated with reduced neuroinflammation, improved barrier integrity, and more homeostatic glial phenotypes, whereas Western-style diets tend to exacerbate immune activation and barrier dysfunction (Kliewer and Mangelsdorf, 2015; Yano et al., 2015; Rowland et al., 2018; Hamamah et al., 2022; Coretti et al., 2024). Importantly, the biological impact of microbial metabolites is context dependent (Table 1). This complexity is particularly evident in tryptophan metabolism, where distinct microbial-derived indoles can either suppress or exacerbate neuroinflammation through AhR signaling, underscoring the need to move beyond simplistic “beneficial versus harmful” classifications (Sittipo et al., 2022; Ashique et al., 2024; Coretti et al., 2024).

Among diet-derived bioactives, cruciferous vegetables, and sulforaphane in particular, offer a uniquely tractable model for studying diet-microbiota-brain interactions. The glucosinolate-to-isothiocyanate conversion pathway tightly couples dietary exposure to microbial and enzymatic bioactivation, converging on the Nrf2 antioxidant and cytoprotective signaling network (Zhao et al., 2018; Gruenbaum et al., 2024; Qu et al., 2024; Bessetti and Litwa, 2025). Evidence from *in vitro* systems, animal models, and early-stage human studies have shown that sulforaphane has the capacity to attenuate oxidative stress, modulate microglial activation, preserve barrier integrity, and influence neurobehavioral outcomes (Zhao et al., 2018; Hills et al., 2019; Patterson et al., 2019; Miri et al., 2023; Braga et al., 2024; Gruenbaum et al., 2024; Bessetti and Litwa, 2025). While sulforaphane should not be viewed as a stand-alone therapeutic, it illustrates how defined dietary substrates can engage conserved host defense pathways through microbiota-dependent mechanisms, providing a useful framework for translational investigation.

Microbiota-targeted interventions, including probiotics, prebiotics, fecal microbiota transplantation (FMT), and anti-inflammatory pharmaceuticals, further support the concept that modifying gut ecosystems can influence neuroinflammatory trajectories. Preclinical and limited clinical studies suggest that such approaches may reduce neuroinflammation, restore barrier function, and improve cognitive or behavioral outcomes in conditions such as AD, PD, MS, and ASD (Gershon and Margolis, 2021; Yadav et al., 2022; Warren et al., 2024; Moretti et al., 2025). Although promising, therapeutic responses remain variable, reflecting interindividual differences in host genetics, immune state, and microbial ecology (Gershon and Margolis, 2021; Warren et al., 2024).

Despite substantial progress, key challenges remain in translating this growing body of work into effective clinical strategies (Box 1). Much of the current evidence derives from animal models or small, heterogeneous human trials (Picciotto et al., 2012; Hamamah et al., 2022; Coretti et al., 2024; Jaberi et al., 2024). Future research should prioritize longitudinal, well-controlled human studies that integrate dietary intake, microbial function, metabolomics, immune profiling, and neuroimaging to establish causal links and clinically actionable biomarkers. Emphasis on functional microbial outputs will be essential for identifying robust biomarkers and therapeutic targets capable of informing personalized interventions.

Collectively, the findings reviewed here support a framework in which diet acts as a primary upstream regulator of microbiota-mediated signaling along the GBA, with meaningful implications for neuroinflammatory and neurodegenerative disease risk and progression (Ma et al., 2019; Ojeda et al., 2021; Bostick et al., 2022; Jaberi et al., 2024; Loh et al., 2024; Yang et al., 2024). Leveraging this framework to develop personalized, mechanism-informed dietary and microbiota-targeted interventions may offer a scalable and complementary approach to existing therapies, making sure that biological complexity, context dependence, and individual variability are accounted for.

## Glossary

GBA: gut-brain axis
GI: gastrointestinal
CNS: central nervous system
ENS: enteric nervous system
PD: Parkinson’s disease
AD: Alzheimer’s disease
MS: multiple sclerosis
ASD: autism spectrum disorder
BBB: blood–brain barrier
SCFAs: short-chain fatty acids
BA/BAs: bile acid(s)
TLRs: toll-like receptors
LPS: lipopolysaccharide
NF-kB: nuclear factor kappa B
HDAC: histone deacetylase
Nrf2: nuclear factor erythroid 2-related factor 2
HO-1: heme oxygenase 1
FXR: farnesoid X receptor
TGR5: takeda G protein-coupled bile acid receptor 1
FFAR2/FFAR3: free fatty acid receptor ⅔
GLP-1: glucagon-like peptide-1
GABA: gamma-aminobutyric acid
TPH1: tryptophan hydroxylase 1
IBD: inflammatory bowel disease
IBS: irritable bowel syndrome
AhR: aryl hydrocarbon receptor
IPANs: intrinsic primary afferent neurons
MMKD: Mediterranean ketogenic diet
EAE: experimental autoimmune encephalomyelitis
NSCs: neural stem cells
CSDS: chronic social defeat stress
BDNF: brain-derived neurotrophic factor
TrkB: tropomyosin receptor kinase B
AITC: allyl isothiocyanate
Mox phenotype: oxidative stress-responsive microglial phenotype
FMT: fecal microbiota transplantation
ALS: Amyotrophic Lateral Sclerosis
HIF: hypoxia-inducible factor
TTC: 2,3,5-triphenyltetrazolium chloride
CCA/MCA: carotid artery/middle cerebral artery
ARE: antioxidant response element
HIE: hypoxic–ischemic encephalopathy
